# A Cross-sectional Survey and Cross-sectional Clinical Trial to Determine the Prevalence and Management of Eye Movement Disorders and Vestibular Dysfunction in Post-Stroke Patients in the Sub-Acute Phase: Protocol

**DOI:** 10.3389/fneur.2016.00140

**Published:** 2016-09-20

**Authors:** Andoret van Wyk, Carina A. Eksteen, Piet J. Becker, Barbara M. Heinze

**Affiliations:** ^1^Department of Physiotherapy, Faculty of Health Sciences, University of Pretoria, Pretoria, South Africa; ^2^Faculty Research Office, Faculty of Health Sciences, University of Pretoria, Pretoria, South Africa; ^3^Department of Speech-Language Pathology and Audiology, Faculty of Humanities, University of Pretoria, Pretoria, South Africa

**Keywords:** stroke, visual impairment, eye movement disorders, vestibular dysfunction, rehabilitation, physiotherapy, prevalence

## Abstract

**Introduction:**

Visual impairment, specifically eye movement disorders and vestibular dysfunction may have a negative influence on the functional recovery in post-stroke patients. This type of sensory dysfunction may further be associated with poor functional outcome in patients’ post-stroke.

**Methods:**

In phase 1, a cross-sectional survey (*n* = 100) will be conducted to determine the prevalence of eye movement disorders and vestibular dysfunction in patients who sustained a stroke. A cross-sectional clinical trial (*n* = 60) will be conducted during phase 2 of the study to determine the effect of the combination of vestibular rehabilitation therapy (VRT) and visual scanning exercises (VSE) (experimental group) integrated with task-specific activities compared with the effect of task-specific activities as an intervention (control group) on patients who present with eye movement impairment and central vestibular dysfunction post-stroke. An audiologist will assess (a) visual acuity (static and dynamic), (b) nystagmus, (c) saccadic eye movements, (d) smooth pursuit eye movements, (e) vestibulo-ocular reflex, and (f) saccular, utricular, and vestibular nerve function. An independent physiotherapist will assess (1) cognitive function, (2) residual oculomotor visual performance, (3) visual–perceptual system, (4) functional balance, (5) gait, (6) functional ability, (7) presence of anxiety and/or depression, and (8) level of participation in physical activity.

**Ethics and dissemination:**

Ethics approval has been obtained from the Ethics Committee of the Faculty of Health Sciences at the University of Pretoria (UP) (374/2015). The study will be submitted as fulfillment for the PhD degree at UP. Dissemination will include submission to peer-reviewed professional journals and presentation at congresses. Training of rehabilitation team members on the integration of VSE and VRT into task-specific activities in rehabilitation will be done if the outcome of the experimental group’s functional performance is clinically and statistically significantly better than the control group on the Barthel Index.

**Trial Registration:**

Pan African Clinical Trials Registry (PACTR201509001223262).

## Introduction

A recent Cochrane review of interventions for eye movement disorders in post-stroke patients concluded that “eye movement disorders may affect over 70% of stroke patients” (1). Eye movement disorders may result in (a) “difficulty maintaining the normal ocular position” (1) and (b) “difficulty moving the eyes appropriately” (ocular motility) (1). Difficulty in “maintaining the normal ocular position” may result in disconjugate eye movement (eyes do not move in the same direction and by equal amount) (1, 2); strabismus (one eye or both eyes deviate so the eyes are no longer aligned); and/or nystagmus (frequent involuntary oscillations and other random eye movements) (1). Difficulty with spontaneous and voluntary movement of the eyes appropriately during functional activities may include impairment of (i) saccadic eye movements (1) [rapid eye movements (400°–600°/s) that shifts the eyes from one target to another] (1, 2), (ii) smooth pursuit eye movements [slow movement of the eyes (1°–30°/s) that serve to keep gaze on a moving visual object of interest when the head is stationary] (3–5), (iii) fixation (ability to maintain a steady eye position on a target) (1), (iv) convergence (the ability to smoothly and automatically turn the eyes in along the midline to observe near objects with single vision) (6), (v) divergence (to turn the eyes outward for single vision of distant objects) (6), (vi) vestibulo-ocular reflex (to ensure stable images on the retina and clear vision during head movements) (1, 2, 7, 8), and (vii) palsy (inability to use the ocular muscles that move the eye horizontally or vertically) (1, 2). Ocular motility and the interpretation of the visual input gathered during eye movements are important for aligning and maintaining the body in space which is fundamental to the successful performance of functional tasks in any particular environment (9).

The anatomical location of the lesion following a hemispheric, subcortical, brainstem or cerebellar stroke may result in the following symptoms (2): (a) posterior parietal cortex [unilateral spatial neglect (USN); impaired eye movements to the contralesional hemifield], (b) posterior occipito-temporal cortical areas (visual field defect; homonymous hemianopia; abnormal eye movement into the hemianopic visual field; USN), (c) frontal lobe (saccadic eye movement and smooth pursuit eye movement dysfunction), (d) basal ganglia [impaired saccadic- and smooth pursuit eye movements; impaired convergence; USN (visual and auditory neglect)], (e) thalamus (conjugate gaze deviation contralateral to the side of the lesion; abnormal subjective visual vertical; inaccurate saccadic eye movements; USN), (f) brainstem (conjugate gaze abnormalities), and (g) cerebellum (impaired smooth pursuit eye movements and VOR suppression; gaze-holding dysfunction; ataxia of the head, trunk, and limbs) (2).

Visuomotor deficits and visual–perceptual impairments as result of eye movement disorders may affect an individual’s ability to respond to sensory input obtained from the environment and demands of the task. The inability to respond efficiently upon the sensory input from the environment and demands of the task results in a change (decrease) in postural control that leads to increased functional dependence during ADL and disability (6, 10). Clinical observations of patients with eye movement disorders following a stroke may include the following (1, 6): (1) head turn or head tilt during near (close-up) tasks, (2) avoidance of near (close-up) tasks, (3) seems to look past the observer, (4) closing or covering one eye during conversations and/or activities due to blurred vision or double-vision, (5) squinting, (6) rubs eyes a lot, (7) having difficulty maintaining eye contact, (8) neglecting one side of the body or space during functional activities, (9) bumping into walls or objects during walking or when maneuvering in a wheelchair, (10) appears to misjudge distance due to a loss of depth perception, (11) difficulty with activities of daily living due to poor eye-hand coordination – knocking objects over or missing objects during reaching, (12) “under reaching” or over reaching for objects, (13) decreased attention during conversations and/or activities (patient “day dreams”), (14) losing the place when reading, (15) having difficulty “seeing” with or without glasses, (16) letters jumping around on the page during reading, (17) experiencing eye strain or headaches, (18) portions of the page being missing when reading, (19) objects or portions of objects not being observed, (20) not seeing people or objects approaching suddenly from one side, and (21) having difficulty concentrating on tasks.

Although the association between visual impairment and impaired functional ability is recognized, “the services available to patients with visual impairment following stroke are inconsistent at present” (1). A significant proportion of patients following a stroke may present with unrecognized eye movement disorders and thus receive minimal management of their visual impairment during rehabilitation that has a wide-ranging impact on their functional performance (1, 11). The authors of the Cochrane review (1) found only two studies that investigated interventions for eye movement disorders; both of these randomized controlled trials investigated the effect of pharmacological intervention and not rehabilitation. The authors in both studies concluded that “there is insufficient evidence to reach a conclusion regarding the effectiveness of the different interventions for patients with eye movement disorders after stroke” (1). Pollock et al. (1) also considered possible management of patients with eye movement disorders that included (1) restitutive intervention that includes saccadic eye movement, smooth pursuit eye movement, and convergence training, (2) compensative intervention that consists of training of eye movements for reading, compensatory head movements, and training in ADL, and (3) standardized diagnostic assessment and management of the visual impairment following the stroke (1).

Results of a matched-pair randomized controlled trial conducted by Van Wyk et al. (12) indicated that visual scanning exercises (VSE) with saccadic eye movement training during task-specific activities for patients with USN following a stroke resulted in significant improvement on impairment and functional activity level after 4 weeks of rehabilitation. However, the authors realized that although the intervention focused on the visual system (VSE integrated with task-specific activities), the visual system also functions in intimate integration with the vestibular system and the somatosensory (proprioceptive, cutaneous, and joint receptors) systems in maintaining postural orientation and stability during functional movement.

Patients with vestibular dysfunction experience symptoms may include (1) visual blurring with head movement, (2) subjective complaints of dizziness and/or imbalance, (3) postural instability, (4) balance dysfunction, (5) increased sway in standing, (6) dysregulated gait, (7) falling, and (8) fear of falling as a result of impaired vestibulo-spinal reflexes, vestibulo-ocular reflexes (VORs), and abnormally activated vestibulo-thalamo-cortical pathways that may result in functional impairments in daily life (13–16). Various interventions that include (a) virtual reality, (b) vibrotactile feedback, (c) optokinetic stimulation, (d) VOR adaptation and saccadic exercises, and (e) gait and balance training have shown to decrease dizziness and improve postural control in patients with peripheral and central vestibular disorders (14). Although vestibular rehabilitation therapy (VRT) is indicated in patients with central and peripheral vestibular dysfunction (14), it is recommended that “enhanced engagement with physical, cognitive, and social stimulation that include novel and multi-modal stimulation result in better general functional outcomes” (13). Another recommendation is that VRT should be patient dependent and progression of the exercises should take the individual patient’s sensorimotor, cognitive, and emotional profiles into account (13, 14, 16).

Growing evidence exists that the central nervous system has the ability to compensate for vestibular impairment and re-weight sensory information (16). Although a recent Cochrane review (17) and other literature (16, 18) reported moderate to strong evidence to support VRT for persons with vestibular dysfunction, the assessment and management of central vestibular dysfunction are not integrated in standard post-stroke rehabilitation but occasionally treated as a separate problem. Limited evidence on the prevalence of (a) visual impairments specifically eye movement disorders and (b) central vestibular dysfunction in patients post-stroke has been identified in the literature. The researcher therefore identified a need to investigate the prevalence of specifically eye movement disorders and central vestibular dysfunction in the sub-acute post-stroke population. Identification of possible presence of visual impairments and central vestibular dysfunction in the post-stroke population may facilitate the development of evidence-based treatment for patients with visual impairment and central vestibular dysfunction following stroke.

There is further a lack of research evidence on the combination of VRT and VSE as part of, and integrated with task-specific activities (the gold standard in post-stroke rehabilitation), on body impairment level, functional activity, and participation levels in the treatment of post-stroke patients. The long-term effect of VSE and VRT integrated with task-specific activities on patients’ activities related to participation also needs to be investigated.

## Methods and Analysis

### Study Design

A cross-sectional survey will be conducted during phase 1 of the study to determine (1) the prevalence of visual impairments and central vestibular dysfunction in patients who are in the sub-acute phase post-stroke and (2) whether there is an association between the prevalence of visual impairments and central vestibular dysfunction in sub-acute post-stroke patients’ (a) cognitive function, (b) residual oculomotor visual performance, (c) visual–perceptual system, (d) functional balance, (e) gait, (f) functional ability, and (g) presence of anxiety and/or depression. The STrengthening the Reporting of OBservational studies in Epidemiology (STROBE) checklist will be used when reporting the results of the cross-sectional survey.

A cross-sectional clinical trial will be conducted during phase 2 of the study to determine the effect of the combination of VRT and VSE integrated with task-specific activities received by patients (Group 1) in the sub-acute phase post-stroke compared with patients who received task-specific activities alone (Group 2) on their: (a) visual impairments, (b) central vestibular dysfunction, (c) cognitive function, (d) visual–perceptual function, (e) residual oculomotor visual performance, (f) functional balance, (g) functional ability, (h) gait, and (i) level of anxiety and/or depression. The duration of the intervention period will be 2 weeks. The long-term effect of the two interventions on patients’ participation in physical activity 20 weeks after rehabilitation has been terminated will also be determined. The CONSORT statement will be used when reporting the results of the cross-sectional clinical trial.

### Research Setting

The study will be conducted at private and public rehabilitation institutions in Pretoria and Johannesburg, Gauteng, South Africa. Patients will be referred by multiple acute private and public health-care settings to the appropriate rehabilitation centers.

### Study Population

All patients who sustained a cerebral vascular incident (CVI), who are in the sub-acute phase post-stroke, and who will be admitted at one of the rehabilitation settings in Pretoria and Johannesburg, Gauteng, South Africa will be eligible to participate in the study. A CVI is defined as “the presence of a focal neurological defect due to vascular cause that is present 24 h” and results in varying degrees of dysfunction in activities of daily living (19). The sub-acute phase post-stroke is defined as the period of time since a patient is physiologically stable to 6 months post-stroke (19).

### Eligibility Criteria: Inclusion Criteria

Inclusion criteria for participants in the first phase of the trial will entail:

(a) Patients who suffered either an ischemic or hemorrhagic stroke (20) clinically diagnosed by a medical specialist (neurologist). Information on the type of stroke will be obtained from the patients’ medical records.
(b) Male and female patients in the age group of 19–84 years (21).
(c) Minimum Mini-Mental State Examination (MMSE) score of 7 (22).
(d) Patients in the sub-acute phase following the stroke who are able to follow instructions (23) and have the capacity to provide informed consent (19).

In phase 2 of the study, patients will have in addition to these criteria also have to be diagnosed with visual impairments and/or central vestibular dysfunction.

### Eligibility Criteria: Exclusion Criteria

Patients with the following characteristics will be excluded from the trial:

(a) Severe dementia, identified by implementing the MMSE (22).
(b) History of an organic disorder or major psychiatric impairment (20).
(c) Other comorbid disease or disability such as cancer or amputation that will prevent or limit assessment of the patients and their, participation or follow-up over a period of 20 weeks (20, 23).
(d) Positive Dix–Hallpike test (to exclude BPPV of posterior and anterior SCCs).
  Because the Dix–Hallpike test and CRT (Epley) maneuver require the head rotation of 45° and the extension of 20° to 30°, patients with a history of neck surgery, recent neck trauma, severe rheumatoid arthritis, atlanto-axial and occipito-atlantal instability, cervical myelopathy or radiculopathy, carotid sinus syncope, Chiari malformation, and vascular dissection syndromes will be excluded from the study (24).
(e) Positive Roll test (to exclude BPPV of horizontal SCCs).
(f) Participation in other pharmacological or rehabilitation intervention studies which can confound the results of this study (20).

In phase 2 of the study, the same inclusion/exclusion criteria will be applicable as in phase 1 since patients will be randomly recruited from patients assessed in phase 1 of the study.

### Sample Size

The sample size of phases 1 and 2 are interrelated and was calculated simultaneously with the assistance of a statistician. All patients who suffered a stroke and will be admitted at one of the pre-selected rehabilitation hospitals in Pretoria and Johannesburg, Gauteng for rehabilitation in the sub-acute phase will be assessed to determine if they meet the inclusion and exclusion criteria of the study. Recruitment and assessment of patients will be done in each of the rehabilitation centers in a consecutive sequence based on the number of patients in a particular center at a given time until an adequate and representative sample group (*n* = 100) for the trial has been reached. An adequate and representative sample size required for phase 1 of the study was calculated based on a relatively small, but similar study (12). These authors reported the geometric mean and 95% confidence interval (CI) at baseline and the end of each week of the treatment period. The expected geometric mean of the Barthel Index value at onset was 45 (12) and an improvement in the change from baseline at 2 weeks for the combination of VSE integrated with task-specific activities group (experimental group) compared with the task-specific activities group (control group) is 36 which is 0.214 on the logarithmic (base 10) scale. From the reported 95% CI for the geometric mean, a SD of 0.179 on the log scale was calculated. Since a change from baseline of interest, this SD was inflated by a factor (√2 to 0.249). Based on this information, a sample of at least 30 patients per group will have 90% power to detect the clinical relevant change when testing at the 0.05 level of significance. If testing was one-sided (VRT, VSE, and task-specific activities), based on the published results of the previous study (12), a sample of 30 patients per group will be required. Hence, to achieve a sample size of 30 patients per group, the cross-sectional part of the study (phase 1) will need to enrol at least 100 patients in the sub-acute phase. Prevalence of visual impairments and central vestibular dysfunction will be of interest. As the latter is expected to be in excess of 70% the above sample size is adequate to estimate the prevalence’s to an accuracy within 10%. One hundred patients who meet the eligibility criteria and will give written informed consent to participate in the study will therefore be included in phase 1 of the trial.

### Data Collection Procedure

#### Cross-Sectional Survey: Phase 1 of the Study

One hundred (*n* = 100) patients, who meet the eligibility criteria, in the research settings will be recruited for possible participation in the study. Data collection in phase 1 will be done at each of the research settings for an estimated period of 4 weeks/rehabilitation setting. Each patient who meets the eligible criteria for phase 1 in a particular setting and who will give informed consent after a thorough explanation of what the study will entail will be included into the study and undergo the battery of audiological and physiotherapy assessment procedures (Table 1). The patients will also be informed that participation in the trial is voluntary and they are free from undue coercion. In this study, the International Classification of Functioning, Disability and Health (ICF) (25) is used as the model of disablement within which the patients’ levels of impairment, functional activity, and participation will be determined and documented. Collection of comprehensive demographic information relating to age, gender, race, affected side post-stroke, dominant side prior to stroke, stroke type, type of residence, level of education, and type of work will be collected from patients. Thereafter, an audiologist will assess (a) visual acuity (static and dynamic), (b) nystagmus, (c) saccadic eye movements, (d) smooth pursuit eye movements, (e) vestibulo-ocular reflex, and (f) saccular, utricular, and vestibular nerve function. A physiotherapist who is not involved in the treatment of these patients will assess (1) cognitive function, (2) residual oculomotor visual performance, (3) visual–perceptual system, (4) functional balance, (5) gait, (6) functional ability, and (7) presence of anxiety and/or depression in these patients. If a specific eye condition or eye disease is suspected, the patient will be referred to an ophthalmologist for ophthalmologic assessment and management. The battery of valid and standardized outcome measures that will be completed by a qualified audiologist and physiotherapist (who will not be involved in the allocation of the patients to the experimental and control groups nor the intervention) is summarized in Table 1. The qualified audiologist and physiotherapist will complete the battery of outcome measures over a 2-day period to minimize possible performance fluctuations due to fatigue as result of the lengthy test protocol.

**Table 1:** **The battery of valid and standardized outcome measures that will be completed by a qualified audiologist and physiotherapist**.

Outcome measure used by the physiotherapist	Objective (phase 1)	Objective (phase 2)	Validity and reliability of outcome measure
Video nystagmography (VNG)	To determine the prevalence of visual impairments and central vestibular dysfunction in patients who sustained a stroke measured by the assessment of (a) nystagmus, (b) saccadic eye movements, and (c) smooth pursuit eye movements	To determine the effect of the combination of VRT and VSE integrated with task-specific activities received by patients in Group 1 compared with patients in Group 2 who received task-specific activities alone on their (a) visual impairments, (b) central vestibular dysfunction, (c) nystagmus, (d) saccadic eye movements, and (e) smooth pursuit eye movements after the intervention period of 2 weeks	Video nystagmography (VNG) records eye movements using digital video image technology to quantify (a) nystagmus, (b) saccadic eye movements, and (c) smooth pursuit eye movements (26)
EyeSeeCam vHIT	To determine the prevalence of visual impairments and central vestibular dysfunction in patients who sustained a stroke measured by the assessment of the vestibulo-ocular reflex	To determine the effect of the combination of VRT and VSE integrated with task-specific activities received by patients in Group 1 compared with patients in Group 2 who received task-specific activities alone on their (a) visual impairments, (b) central vestibular dysfunction, and (c) vestibulo-ocular reflex after the intervention period of 2 weeks	Measurements of the HIT with the EyeSeeCam vHIT quantify the VOR-deficit and detect the presence of corrective saccades (2, 27). The head impulse test presents with sensitivity (84%) and specificity (82%) (2, 27)
Vestibular-evoked myogenic potential (VEMP), cervical vestibular-evoked myogenic potential (cVEMP), and ocular vestibular-evoked myogenic potential (oVEMP)	To determine the prevalence of visual impairments and central vestibular dysfunction in patients who sustained a stroke measured by the assessment of the saccular (cVEMP), utricular (oVEMP), and vestibular nerve function	To determine the effect of the combination of VRT and VSE integrated with task-specific activities received by patients in Group 1 compared with patients in Group 2 who received task-specific activities alone on their (a) visual impairments, (b) central vestibular dysfunction, and (c) saccular, utricular, and vestibular nerve function after the intervention period of 2 weeks	Intraclass correlation coefficient (ICC) values ranged between 0.78 and 0.96 and CV_ME_ values ranged from 4 to 36% for P1 and N1 latency, threshold, and interpeak amplitude (28). ICC values of 0.65–0.68 and CV_ME_ values of 170–189% were obtained for the asymmetry ratio (28)
LogMar chart – static visual acuity	To determine the prevalence of visual impairments and central vestibular dysfunction in patients who sustained a stroke measured by the assessment of visual acuity (static)	To determine the effect of the combination of VRT and VSE integrated with task-specific activities received by patients in Group 1 compared with patients in Group 2 who received task-specific activities alone on their (a) visual impairments, (b) central vestibular dysfunction, and (c) visual acuity after the intervention period of 2 weeks	The LogMar chart that follows the principle of logarithmic size progression during the assessment of visual acuity is “considered to be the gold standard for the assessment of distant vision” (29). No differences were noted between test and retest measurements of aided visual acuity (*t* = −0.048, *p* = 0.96) or unaided visual acuity (*t* = −0.038, *p* = 0.97); therefore, the LogMar chart has no learning effect. Test–retest measures of aided and unaided visual acuity are highly correlated, *r* = 0.72 (*p* = 0.000) and *r* = 0.84 (*p* = 0.000) (30)
LogMar chart – dynamic visual acuity	To determine the prevalence of visual impairments and central vestibular dysfunction in patients who sustained a stroke measured by the assessment of the vestibulo-ocular reflex	To determine the effect of the combination of VRT and VSE integrated with task-specific activities received by patients in Group 1 compared with patients in Group 2 who received task-specific activities alone on their (a) visual impairments, (b) central vestibular dysfunction, and (c) vestibulo-ocular reflex after the intervention period of 2 weeks	Provides an objective, behavioral assessment of vestibulo-ocular reflex (VOR) function in response to rotational head movement stimuli. The Dynamic Visual Acuity test (DVA) assesses visual acuity during head movement relative to baseline static visual acuity assessed by the LogMar chart. Unilateral vestibular hypofunction (UVH) (*n* = 41), 10 with UVH mean age = 49.4 (15.5); range 19–70 years: the frequency of head motion affects clinical DVA scores in patients with UVH. DVA scores significantly decreased as head motion frequency increased from 0.5 to 1.0 Hz and from 1.0 to 1.5 Hz, during horizontal (yaw) and vertical (pitch) head movements with both vision charts (*p* < 0.001). Research evidence suggests that DVA be administered with horizontal and vertical rotational head movements of at least 1.5 Hz with the LogMar chart (31)
Mini-Mental State Examination	To determine the association between the presence of visual impairments and central vestibular dysfunction on the patients’ cognitive function. Patients must score at least 7/30 on this scale to be included into the study	To determine the effect of the combination of VRT and VSE integrated with task-specific activities received by patients in Group 1 compared with patients in Group 2 who received task-specific activities alone on their (a) visual impairments, (b) central vestibular dysfunction, and (c) cognitive function after the intervention period of 2 weeks	The MMSE has significant correlates with the Barthel Index (32) assessing activities of daily living, the Montgomery Asberg Depression Rating Scale (MADRS) (33), and the Zung Depression Scale (34, 35). The internal consistency of the MMSE ranged from (alpha = 0.54–0.96) (36). McDowell et al. (37) reported the internal consistency of the MMSE was adequate (alpha = 0.78) (37). Twenty-four studies reported test–retest reliability (*r* > 0.75) for the MMSE (36)
Vula eye health mobile application	To determine the prevalence of visual impairments and central vestibular dysfunction in patients who sustained a stroke measured by the assessment of visual acuity and screening of the patients’ eye health condition	To determine the effect of the combination of VRT and VSE integrated with task-specific activities received by patients in Group 1 compared with patients in Group 2 who received task-specific activities alone on their (a) visual impairments, (b) central vestibular dysfunction, (c) visual acuity, and (d) eye health condition after the intervention period of 2 weeks	The researcher did not find any publication with regard to the mobile application’s validity and reliability in the stroke population but decided to include this measure because visual acuity and eye health condition are very important visual function to determine because intervention in phase 2 of the study need to take a patient’s visual acuity and eye health condition into consideration when implementing the patient’s intervention
			If a specific eye condition or eye disease is suspected, the patient will be referred to an ophthalmologist for ophthalmologic assessment and management
King-Devick Test © (4)	To determine the association between the presence of visual impairments and central vestibular dysfunction on the patients’ residual oculomotor visual performance	To determine the effect of the combination of VRT and VSE integrated with task-specific activities received by patients in Group 1 compared with patients in Group 2 who received task-specific activities alone on their (a) visual impairments, (b) central vestibular dysfunction, and (c) residual oculomotor visual performance after the intervention period of 2 weeks	The King–Devick (K–D) test is a <2-min bedside test that requires saccadic eye movements for rapid visual performance measurement. Although the test is quick to perform, the researcher did not find any publication with regard to the test’s reliability in the stroke population. Moster et al. (38) reported that total time values were significantly higher compared with the control group (*p* = 0.003). Increased K–D scores were associated with decreased values for binocular low-contrast (*p* < 0.001) and high-contrast visual acuity (*p* = 0.003). It is therefore recommended that the K–D test be included in the battery of valid and standardized outcome measures completed by a qualified physiotherapist during phase 1 and phase 2 of the study
Star cancelation test (4)	To determine the association between the presence of visual impairments and central vestibular dysfunction on the patients’ visual–perceptual function	To determine the effect of the combination of VRT and VSE integrated with task-specific activities received by patients in Group 1 compared with patients in Group 2 who received task-specific activities alone on their (a) visual impairments, (b) central vestibular dysfunction, and (c) visual–perceptual function after the intervention period of 2 weeks	Plummer et al. noted that Marsh and Kersel reported the correlation between the Line Bisection Test with the Star Cancellation Test (Pearson *r* = −0.40, *p* = 0.02) in (*n* = 27) stroke patients (39). The star cancellation test correlated with other clinical tests of unilateral spatial neglect, indicating a construct validity of (Pearson *r* = 0.26–0.78) (39). Bailey et al. reported the sensitivity of the star cancelation test = 76.4% compared with other copying tests (57.5%) (40)
Berg balance scale	To determine the association between the presence of visual impairments and central vestibular dysfunction on the patients’ functional balance (static)	To determine the effect of the combination of VRT and VSE integrated with task-specific activities received by patients in Group 1 compared with patients in Group 2 who received task-specific activities alone on their (a) visual impairments, (b) central vestibular dysfunction, and (c) functional balance after the intervention period of 2 weeks	The Berg Balance Scale (BBS) has an excellent test–retest reliability (ICC = 0.88) (41). The BBS correlated with other functional measurements in various older adults with disability; Barthel Index (Pearson *r* = 0.67, *n* = 31), Fugl-Meyer Test motor and balance subscales (Pearson *r* = 0.62–0.94, *n* = 60), Timed Up, and Go Test (TUG) scores (Pearson *r* = 0.76, *n* = 31) and the Dynamic Gait Index (Spearman coefficient = 0.67, *n* = 44) (42)
Dynamic gait index (DGI) (7, 34)	To determine the association between the presence of visual impairments and central vestibular dysfunction on the patients’ gait (dynamic balance)	To determine the effect of the combination of VRT and VSE integrated with task-specific activities received by patients in Group 1 compared with patients in Group 2 who received task-specific activities alone on their (a) visual impairments, (b) central vestibular dysfunction, and (c) gait after the intervention period of 2 weeks	McConvey and Bennett (43) reported inter-rater reliability (ICC for total DGI = 0.983), with individual test items ranging from 0.910 to 0.976 in patients with MS (*n* = 10). Intrarater reliability ranged from 0.760 to 0.986 and an inverse correlation (−0.801) between gait speed on a 6.1 m course and total DGI score (43). Lin et al. (44) reported good test–retest reliability (ICC > 0.94; 0.91–0.97) in stroke patients (*n* = 48) (44). Jonsdottir and Cattaneo (45) reported good test–retest reliability (ICC = 0.96) and individual items varied from 0.56 (gait and pivot turns) to 1.00 (stair climbing) in chronic stroke patients (*n* = 25) (45)
Barthel index (BI) (4)	To determine the association between the presence of visual impairments and central vestibular dysfunction on the patients’ functional ability	To determine the effect of the combination of VRT and VSE integrated with task-specific activities received by patients in Group 1 compared with patients in Group 2 who received task-specific activities alone on their (a) visual impairments, (b) central vestibular dysfunction, and (c) functional ability after the intervention period of 2 weeks	The Barthel Index’s (BI) calculated inter-rater reliability using the intraclass correlation (ICC) = 0.94 and the internal consistency using Cronbach’s alpha ranges between 0.89 and 0.92. The BI also closely correlated with the Berg Balance Scale and the Fugl-Meyer motor assessment in patients with stroke (Pearson’s correlation coefficient *r* ≥ 0.78) (46)
			Hsueh et al. reported inter-rater reliability for individual items (kappa value range, 0.53–0.94) and total score (ICC = 0.94) of the BI in stroke patients (*n* = 121). The BI correlated with the Fugl-Meyer Motor Assessment (Pearson’s *r* ≥ 0.78, *p* < 0.0001) the Frenchay Activities Index (Pearson’s *r* ≥ 0.59, *p* < 0.0001) (46)
Hospital anxiety and depression scale	To determine the association between the presence of visual impairments and central vestibular dysfunction on the patients’ level of anxiety and depression	To determine the effect of the combination of VRT and VSE integrated with task-specific activities received by patients in Group 1 compared with patients in Group 2 who received task-specific activities alone on their (a) visual impairments, (b) central vestibular dysfunction, and (c) level of anxiety after the intervention period of 2 weeks	Correlation between the HADS and the BDI ranges from *r* = 0.61 to 0.83. Correlation between the General Health Questionnaire, the Clinical Anxiety Scale, and the HADS ranged from *r* = 0.50 to 0.68 and *r* = 0.69 to 0.75, respectively. The HADS presents with an internal consistency with a Cronbach’s alpha of 0.85 (47)
Telephonic-administered international physical activity questionnaire (IPAQ)	To determine the association between the presence of visual impairments and central vestibular dysfunction on the patients’ participation in physical activity	To determine the effect of the combination of VRT and VSE integrated with task-specific activities received by patients in Group 1 compared with patients in Group 2 who received task-specific activities alone on their (a) visual and ocular impairments and (b) central vestibular dysfunction post-stroke’s participation in physical activity 20 weeks after rehabilitation has been terminated	Correlation between the telephonic-administered IPAQ compared with a face-to-face administered IPAQ was found to be 0.92 for the section on leisure-time, and 0.87 for the section on transport-related activities (48). The telephone interview reliability kappa was 0.78 (48)
			Reliability data for the IPAQ short questionnaires (*n* = 1974): repeatability is at an acceptable level, with 75% of the correlation coefficients observed above 0.65 and ranging from 0.32 to 0.88 (49)

#### Cross-Sectional Clinical Trial: Phase 2 of the Study

Patients with or without visual impairment and/or vestibular dysfunction will be identified based on the results of patients’ diagnosis after the battery of standardized outcome measures has been completed by two independent assessors (audiologist and physiotherapist) during phase 1 of the study. Sixty patients with (visual and/or vestibular dysfunction) identified in phase 1 of the study will be recruited to participate in the second phase of the study and randomly allocated to either Group 1 or Group 2. The study population will include the data of the first 30 patients in each group who participate in the study for all 20 weeks. The two groups of 30 patients in the respective group do not make provision for drop out of participants in the study. If patients leave the study for any reason, they will have to be replaced by another patient. The results of this first battery of tests (phase 1) will serve as the baseline measurements for phase 2 of the study. In phase 2, the researcher will determine the effect of the combination of VRT and VSE integrated with task-specific activities (*n* = 30) as an intervention approach for patients in the sub-acute phase post-stroke compared with patients who will receive task-specific activities (*n* = 30) alone as an intervention approach on their: (a) visual impairments, (b) central vestibular dysfunction, (c) cognitive function, (d) residual oculomotor visual performance, (e) visual–perceptual system, (f) functional balance, (g) ability to modify gait in response to changing task demands, (h) functional ability, and (i) presence of anxiety and/or depression after the intervention period of 2 weeks. The consecutive assessment will be repeated by the two independent assessors after the 2-week intervention period. Twenty weeks after rehabilitation has been terminated, the physiotherapist (independent assessor) will contact all patients who completed phase 2 of the study, to complete the Telephonic-Administered International Physical Activity Questionnaire (IPAQ). The aim will be to determine the effect of the combination of VRT and VSE integrated with task-specific activities for patients in Group 1 (experimental group), compared with patients who will receive task-specific activities alone Group 2 (control group) on patients’ participation activities 20 weeks after rehabilitation has been terminated.

Patients will receive intervention daily for a period of 2 weeks. The period of intervention consisting of 2 weeks was selected due to the fact that patients are on average admitted to a private rehabilitation setting post-stroke for a period of 2 weeks. The intervention period of 2 weeks were further decided upon because results from a previous study (12) demonstrated a statistically significant (*p* = 0.02) difference between Group 1 and Group 2 after 2 weeks intervention (12). Any adverse effects within the period of 20 weeks will be documented.

### Interventions

#### Experimental Group

Patients in the experimental group will receive a combination of VRT and VSE integrated with task-specific activities as part of the treatment as an “add on” intervention in this trial. A guide on the principles of VSE integrated with task-specific activities and the principles of progression of these exercises (12) is described in Table 2.

**Table 2:** **Guide of the principles of saccadic eye movement training with visual scanning exercises integrated with task-specific activities and the principles of progression of these exercises (12)**.

Functional positions	Progression of task-specific activity	Visual scanning exercises integrated with task-specific activity	Progression of visual scanning exercises integrated with task-specific activity
Supine	(1) Bridging with feet on a balance mat (i.e., Airex balance mat). ↓ (2) Bridging with feet on a balance ball.	(1) Manually and/or verbally facilitate head rotation toward impaired side. ↓ (2) Perform bridging while doing saccadic eye movements by naming individual letters or numbers aloud on flash cards that are “flashed” by the physiotherapist from all directions. Example: 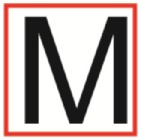 • Flash cards are shown on the impaired side by the treating physiotherapist. ↓ • Flash cards are placed against the wall on the impaired side. ↓ (3) Bridge while doing small saccadic eye movements by naming individual letters aloud on a HART-chart. Example: 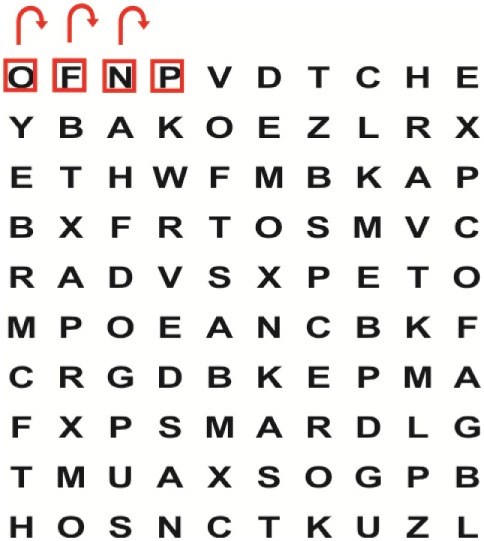	(1) Bridging (lift buttocks up) while naming letter aloud on flash card. Example: 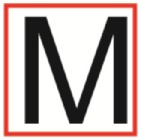 ↓ Return to supine (drop buttocks) and immediately naming letter aloud on flash card. Example: 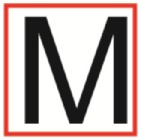 • Repeat activity with various flash cards with different letters/numbers and from different directions. ↓ (2) Bridging (lift buttocks up) while naming letter aloud on the HART-chart. Example: 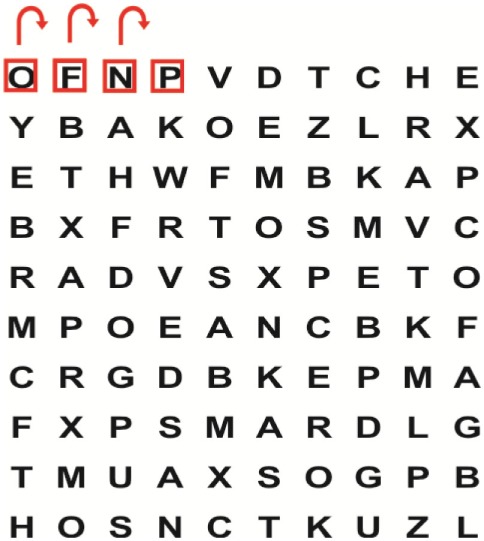 ↓ Return to supine (drop buttocks) and immediately naming the next letter aloud on the HART-chart. 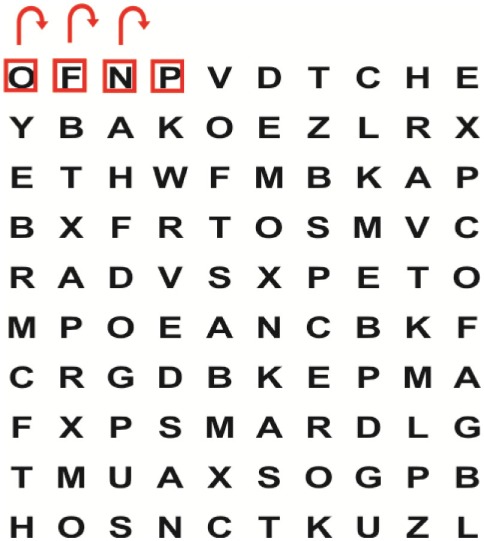 • Repeat activity on all lines of HART-chart. ↓ (3) Bridging (lift buttocks up) while naming letter aloud on the HART-chart from top to bottom. Example: 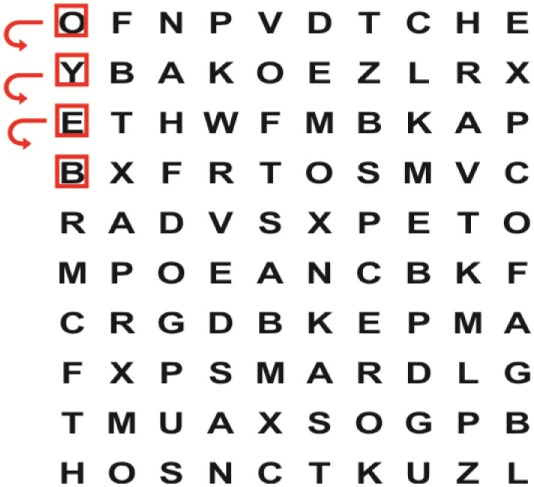 ↓ Return to supine (drop buttocks) and immediately naming the next letter aloud on the HART-chart. Example: 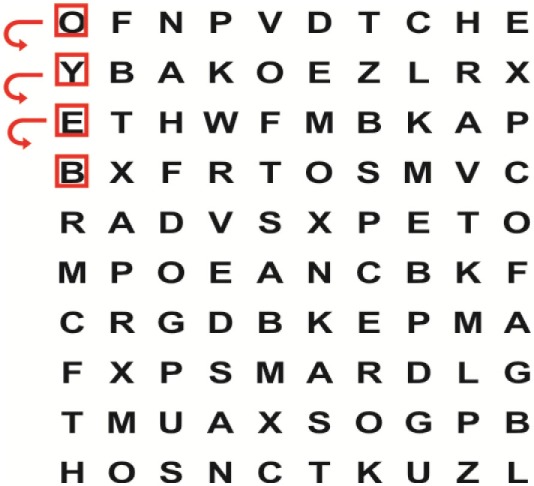 • Repeat activity on all lines of HART-chart.
Side lying to sitting	Side lying to sitting on an uneven surface (i.e., on a soft mat)	Move from supine to side lying and from side lying to sitting while fixating the eyes on a number or letter on a flash card. Example: 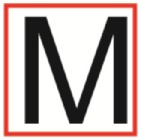	(1) Incorporate smooth pursuit eye movements and visual fixation by tracking of an object: • Patient fixates eyes on an object that is moved by the treating physiotherapist toward the impaired/affected side while sitting up. • Keep eyes fixated on moving object, head may turn while sitting up. • Keep head still while continuing to fixate on an object that is moving toward the impaired/affected side while sitting up.
Sitting	(1) Sitting on a balance mat (2) Sitting on a balance disk (3) Sitting on a roller (4) Sitting on an exercise ball	(1) 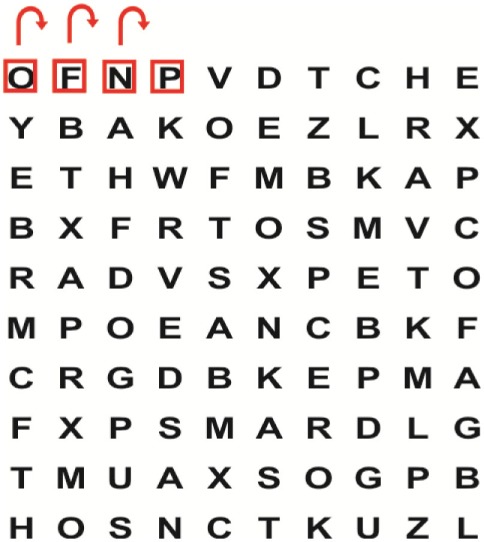 ↓ (2) 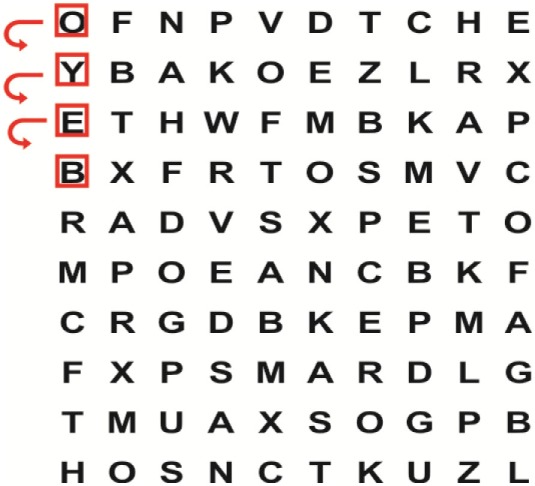	(1) Progress to larger saccadic eye movements and visual search strategies. 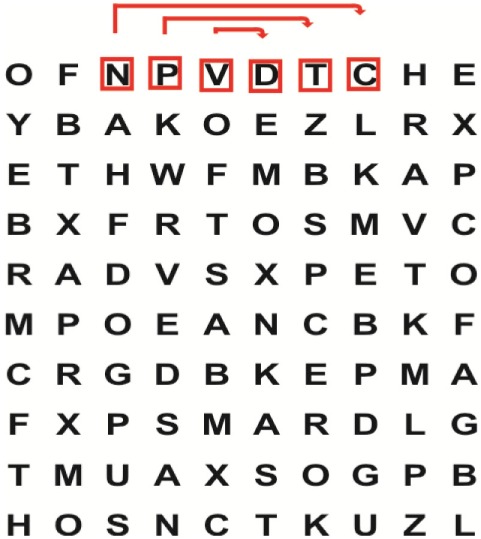 • Start in the middle of the row and progress from (L) to (R). Increase the saccadic eye movements by progressing outwards toward the furthest letter/number on the (L) and (R). • Repeat activity on all lines of HART-chart. ↓ (2) Progress to larger saccadic eye movements and visual search strategies. 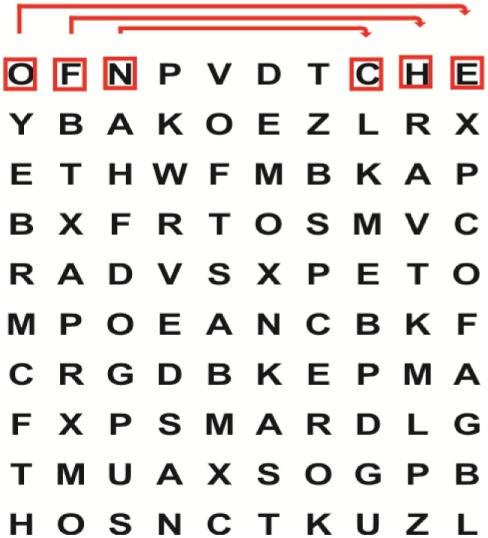 • Name the letter furthest on the (L), “jump” with eyes immediately to the letter furthest on the (R). Repeat the process by naming the second letter on the (L) and immediately the second letter on the (R). Repeat till the middle of the row inwards. • Repeat activity on all lines of HART-chart. ↓ (3) 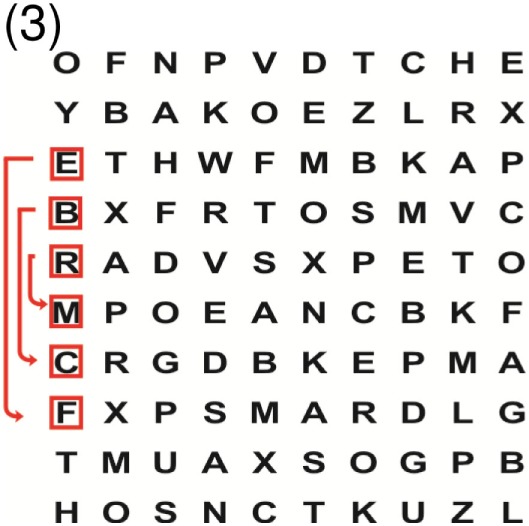 ↓ • Start in the middle of the row and progress from top to bottom. Increase the saccadic eye movements by progressing outwards toward the furthest letter/number on the top and bottom. • Repeat activity on all lines of HART-chart. ↓ (4) 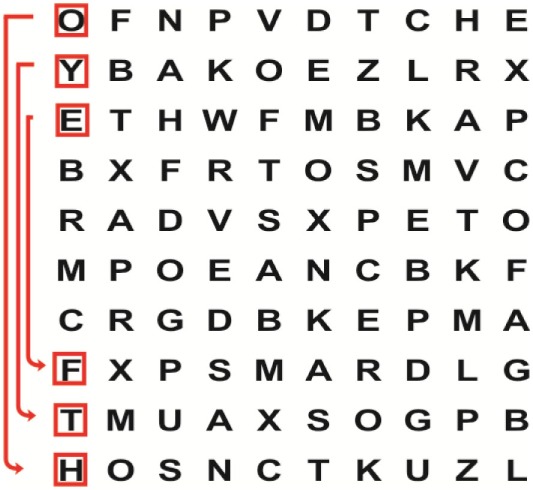 • Name the letter furthest on the top, “jump” with eyes immediately to the letter furthest on the bottom. Repeat the process by naming the second letter on the top and immediately the second letter on the bottom. Repeat till the middle of the row inwards. • Repeat activity on all lines of HART-chart. (5) Incorporate smooth pursuit eye movements and visual fixation by tracking of an object: • Patient fixates eyes on an object that is moved by the treating physiotherapist toward the impaired/affected side while sitting. • Keep eyes fixated on moving object, head may turn while sitting up. • Keep head still while continuing to fixate on an object that is moving toward the impaired/affected side while sitting up.
Sit to stand	(1) With support in front of a table (2) Without support of a table (3) Sit to stand on an even surface (4) Sit to stand on an uneven surface, i.e., balance mat	Move from sit to stand while fixating the eyes on a number or letter on a flash card. Example: 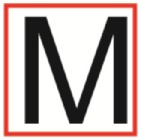	(1) Move from sit to stand while naming letter aloud on a flash card. Example: 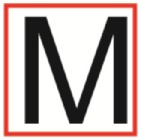 ↓ (2) Move from standing to sitting while naming letter aloud on a flash card. Example: 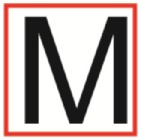 • Repeat activity with various flash cards with different letters. ↓ (3) Move from sit to stand while naming letter aloud on a HART-chart. Example: 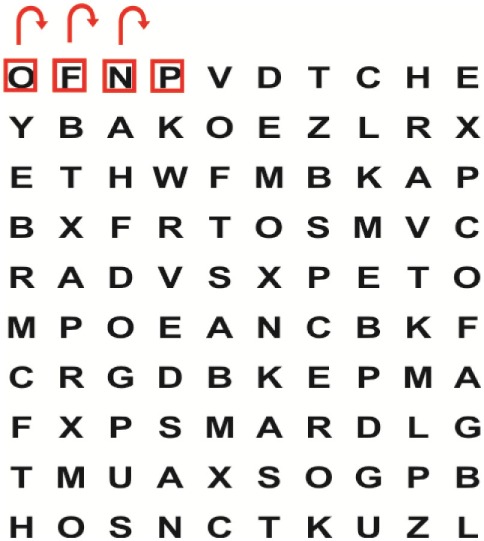 ↓ (4) Move from standing to sitting while naming letter aloud on a HART-chart. Example: 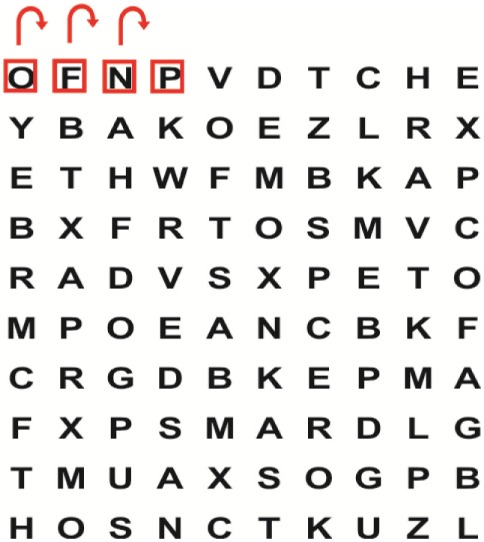 • Repeat activity on all lines of HART-chart. ↓ (5) Move from sit to stand while naming letter aloud on a HART-chart. Example: 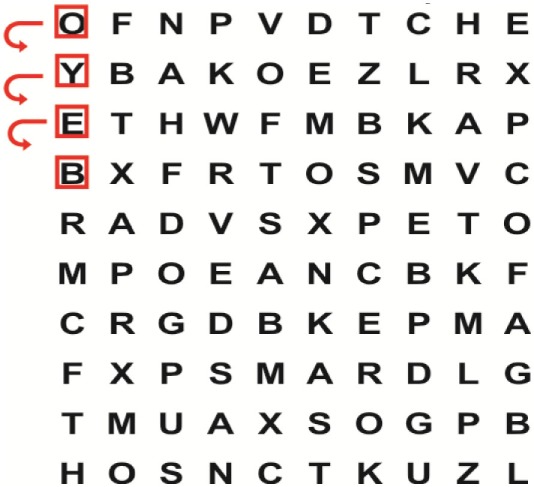 ↓ (6) Move from standing to sitting while naming letter aloud on a HART-chart. Example: 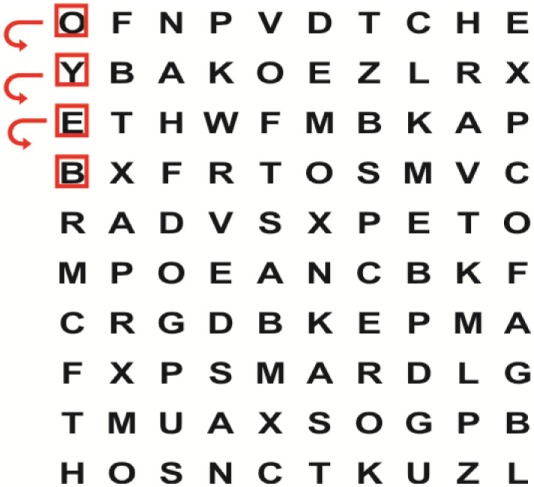 • Repeat activity on all lines of HART-chart.
Standing	(1) With support in front of a table (2) Without support of a table With an assistive device – walking frame; crutch; quadpod; tripod, walking stick (3) Without an assistive device (4) Standing near a wall for support (5) Stand in the middle of a room without support (6) Standing on a proprioception mat (7) Standing on balance disk or Bosu ball (8) Standing on a mini – trampoline	Performing saccadic eye movements with visual scanning exercises while in standing. (1) 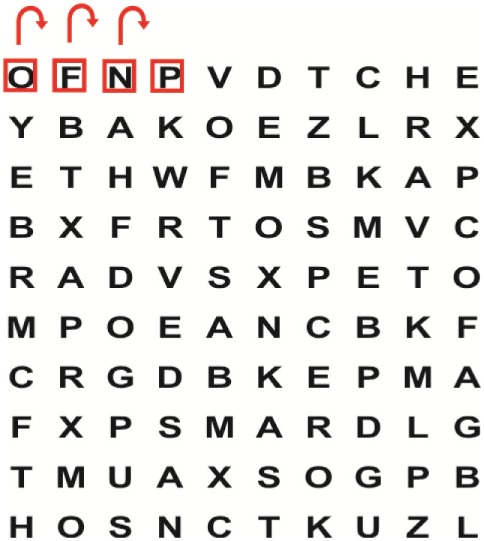 ↓ (2) 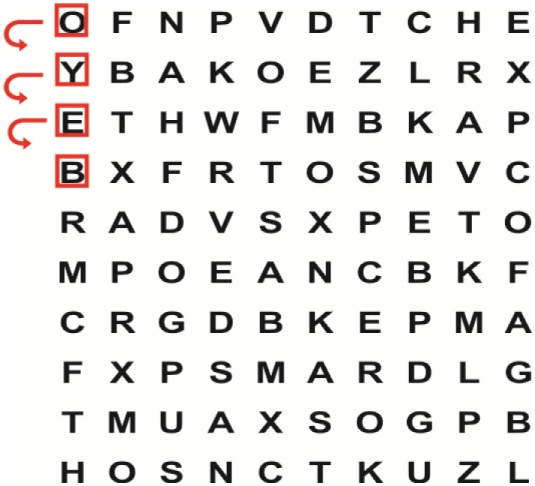	(1) Progress to larger saccadic eye movements and visual search strategies in standing. 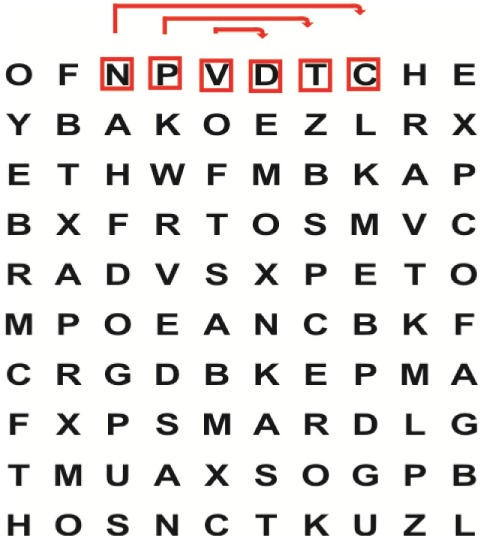 • Start in the middle of the row and progress from (L) to (R). Increase the saccadic eye movements by progressing outwards toward the furthest letter/number on the (L) and (R). • Repeat activity on all lines of HART-chart. ↓ (2) Progress to larger saccadic eye movements and visual search strategies instanding. 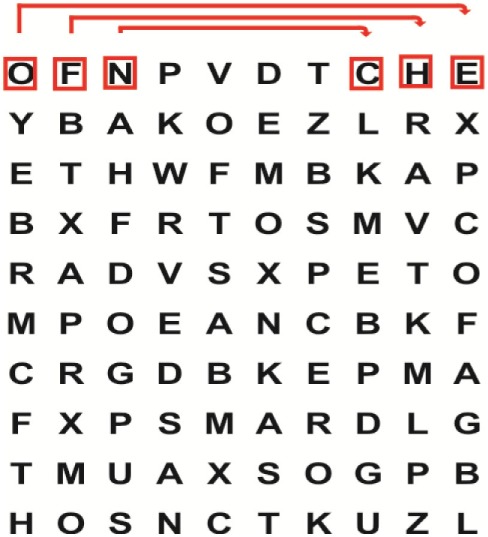 • Name the letter furthest on the (L), “jump” with eyes immediately to the letter furthest on the (R). Repeat the process by naming the second letter on the (L) and immediately the second letter on the (R). Repeat till the middle of the row inwards. • Repeat activity on all lines of HART-chart. ↓ (3) 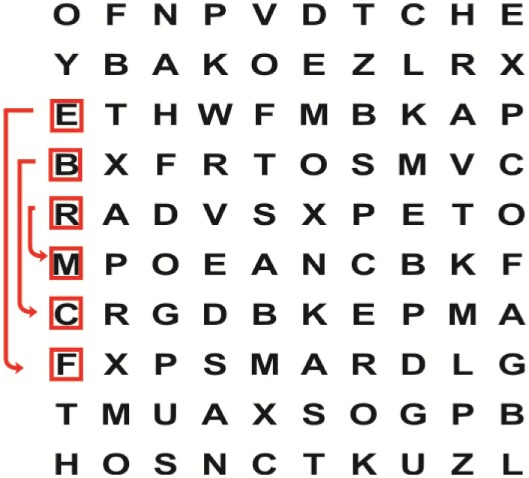 ↓ • Start in the middle of the row and progress from top to bottom. Increase the saccadic eye movements by progressing outwards toward the furthest letter/number on the top and bottom. • Repeat activity on all lines of HART-chart. ↓ (4) 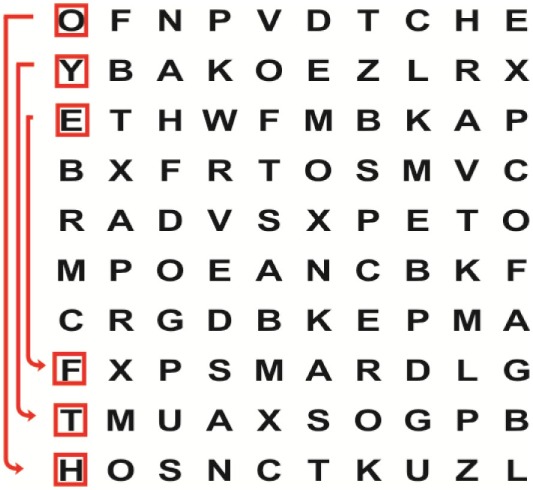 • Name the letter furthest on the top, “jump” with eyes immediately to the letter furthest on the bottom. Repeat the process by naming the second letter on the top and immediately the second letter on the bottom. Repeat till the middle of the row inwards. • Repeat activity on all lines of HART-chart. (5) Incorporate smooth pursuit eye movements and visual fixation by tracking of an object: • Patient fixates eyes on an object that is moved by the treating physiotherapist toward the impaired/affected side while sitting. • Keep eyes fixated on moving object, head may turn while sitting up. • Keep head still while continuing to fixate on an object that is moving toward the impaired/affected side while sitting up.
Half-standing	(1) With support in front of a table (2) Without support of a table (3) With an assistive device – walking frame; crutch; quadpod; tripod, walking stick (4) Without an assistive device (5) Standing near a wall for support (6) Stand in the middle of a room without support (7) One leg on the floor and one leg on a balance mat/Bosu ball (8) One leg on the balance mat and one leg on a step (9) One leg on the balance mat and one leg on a balance ball	(1) Place one leg on a step while performing saccadic eye movements with visual scanning exercises. 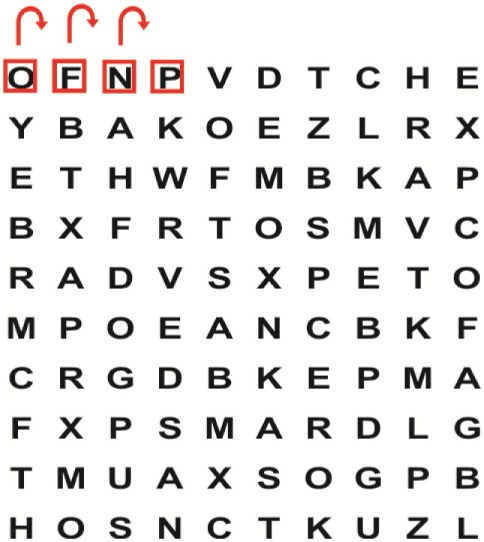 ↓ 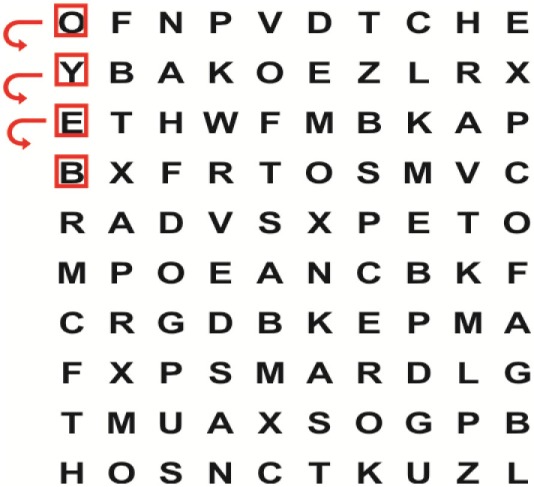 ↓ 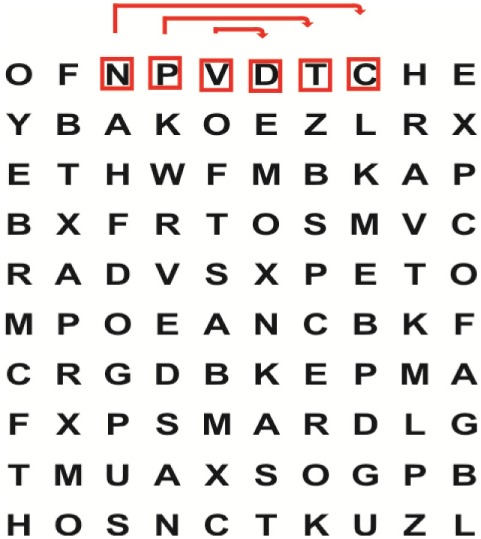 ↓ 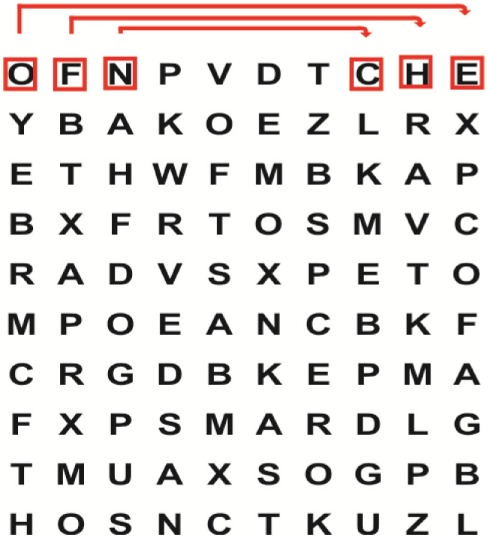 ↓ 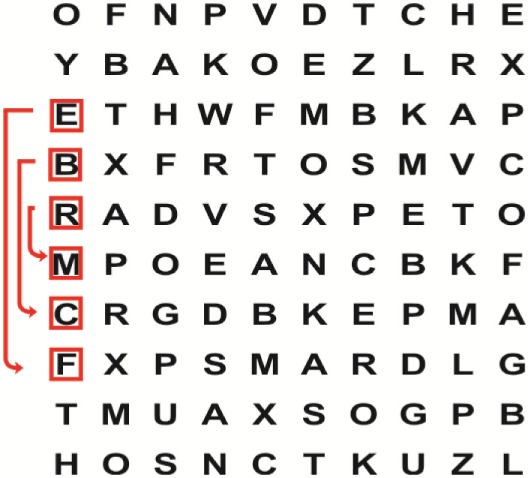 ↓ 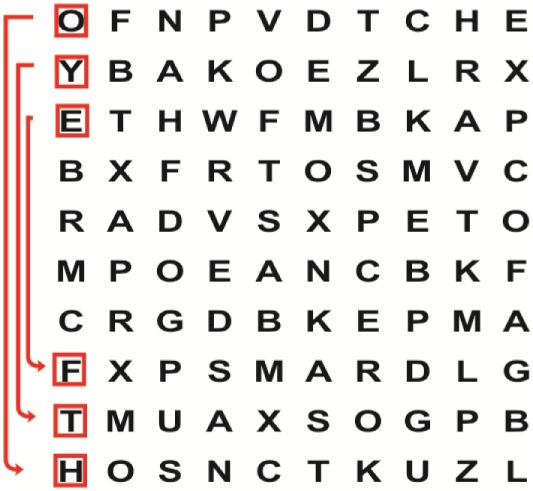	(1) Alternate legs on the step while performing visual scanning exercises. One leg on the floor and one leg on a step. • Place one leg on the step while naming letter aloud on HART-chart. • Alternate legs by placing the other leg and immediately naming aloud the next letter on the HART-chart. (2) Incorporate flash card between naming of letters on HART-chart. Example: 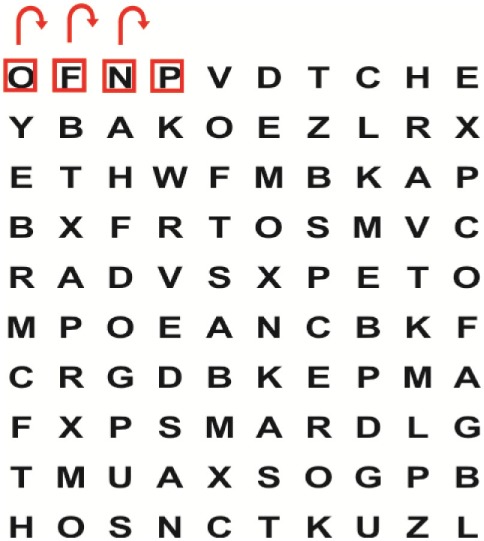 ↓ 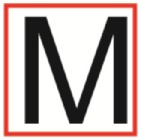 ↓ 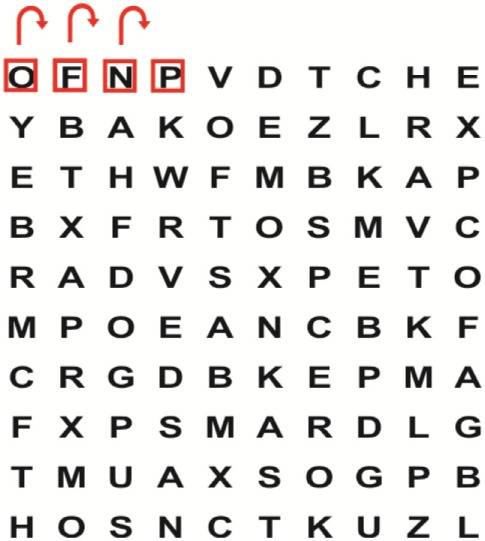 ↓ 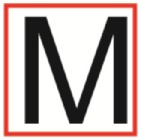 • Repeat activity on all lines of HART-chart.
Gait	(1) With an assistive device – walking frame; crutch; quadpod; tripod, walking stick (2) Without an assistive device (3) While holding a tray (4) Walking with one foot on an AIREX balance beam and the other foot on the floor (even surface) (5) Walking in a figure of eight	(1) Walking on an even/uneven surface while fixating the eyes on a number or letter on a flash card. Example: 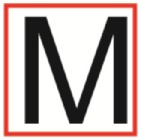 (2) Walking on an even/uneven surface while performing saccadic eye movements with visual scanning exercises. 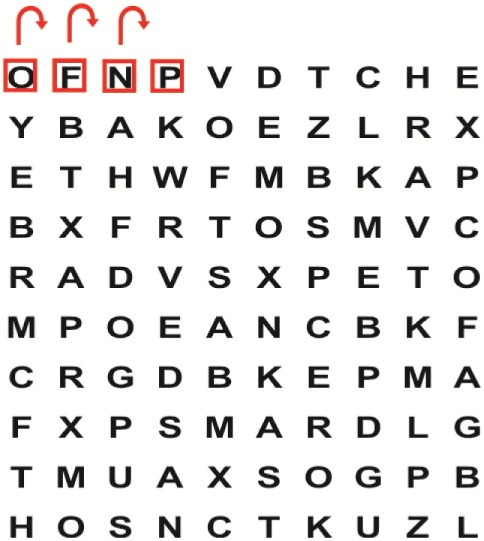 ↓ 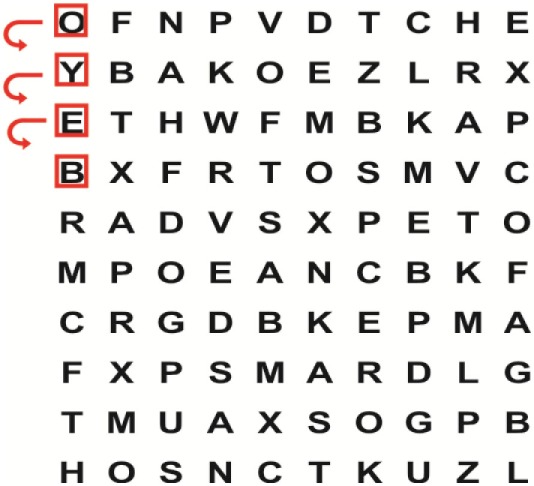 ↓ 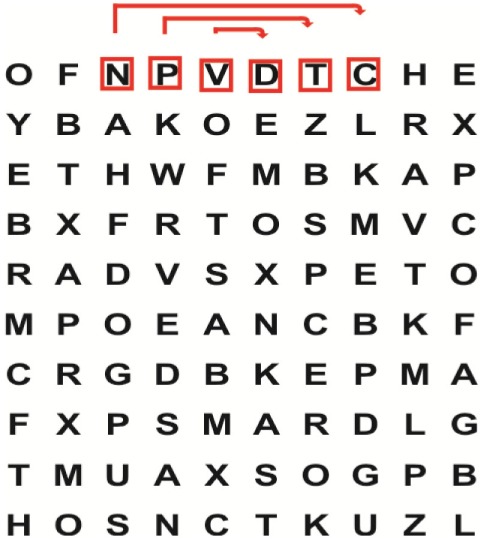 ↓ 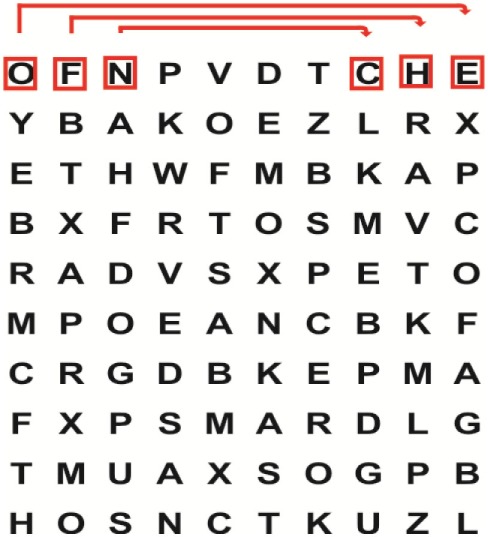 ↓ 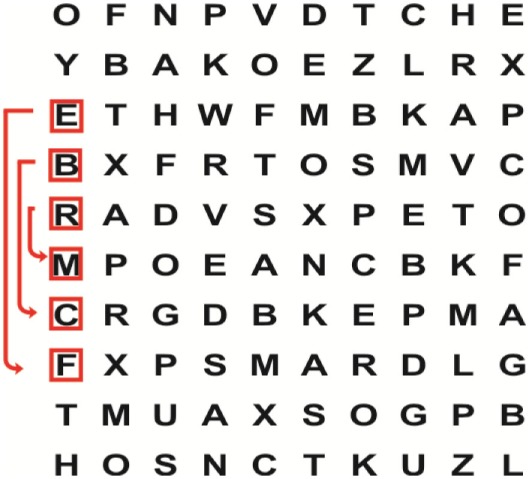 ↓ 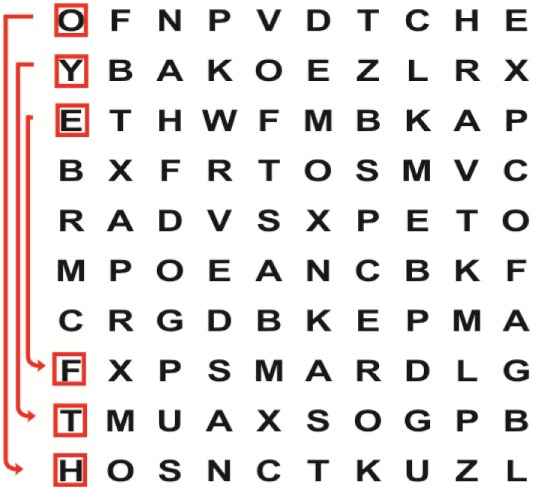	(1) Keep eyes fixated on a flash card while turning. Example: 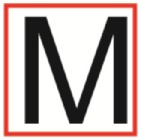 (2) Incorporate flash card between naming of letters on HART-chart. Example: 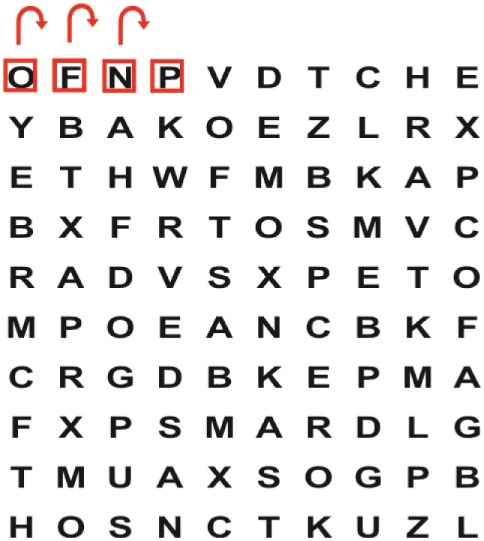 ↓ 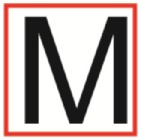 ↓ 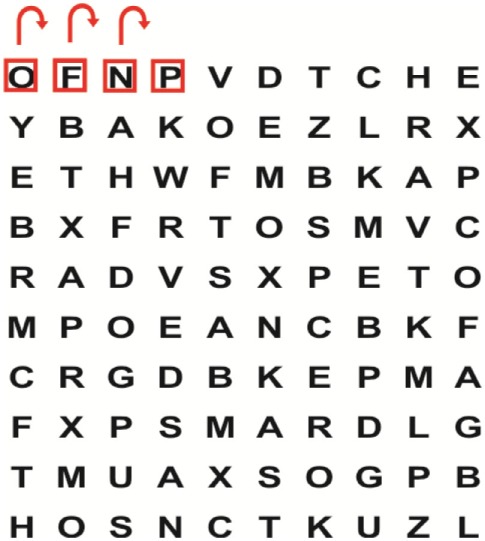 ↓ 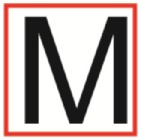 • Repeat activity on all lines of HART-chart. (3) Walking while holding a tray. Placing flash cards on the tray while walking, reading the numbers on the flash cards aloud. Example: 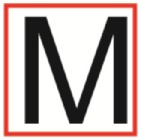

Vestibular rehabilitation therapy for treatment of central vestibular dysfunction consists of a program of exercises designed to (i) facilitate adaptation of the vestibular system, (ii) habituate the person to movement, (iii) teach sensory substitution, and (iv) as such improve a person’s balance and postural control (50).

##### Vestibular Adaptation Exercises

“Vestibular adaptation refers to the long-term changes” that take place in the vestibular system in response to stimuli that induce adaptation (51). The preferred stimuli to facilitate adaptation are to produce an error signal that the CNS attempts to decrease by modifying the gain of the vestibular system (51). Gaze-stabilization exercises to retrain VOR function are prescribed to stimulate retinal slip to optimize vision during head movement and as such to achieve vestibular adaptation. The first exercise consists of having the patient focus on a target while moving their head as fast as they can and still maintain fixation on the target. The exercise is performed in various functional positions, during gait, walking with a different speed, against various backgrounds, at different distances from the target and in different planes of movement (14, 16). The second exercise consists of having the patient focus on a target while the target and the head move in opposite directions while the patient keeps the target in focus by maintaining visual fixation on the target (51). The exercise is performed in various functional positions, during gait, walking with a different speed, against various backgrounds, during different distances and in different planes (14, 16).

##### Habituation Exercises

The purpose of habituation exercises is to facilitate a reduction in symptoms through repetitive exposure and practice of a movement that provokes the symptoms experienced by the patient. The aim of habituation exercises is to “de-sensitize” the patient and to improve motion-induced dizziness or vertigo (18, 50, 51). Positions and movements may include moving from sitting to lying in supine, rolling from supine to left side lying, rolling from supine to right side lying, moving from supine to sitting, moving from sitting to left and right Dix–Hallpike position, return to sitting from the Dix–Hallpike position, horizontal and vertical movement of the head and turning 180° to the left and right (51).

##### Substitution Exercises

Saccadic eye movement substitution is another method that is aimed to decrease visual blurring and dizziness (14). Errors in gaze position were reduced when compensatory saccadic eye movements were utilized as part of the gaze-stabilization process. The combination of decreased retinal slip and saccadic eye movement substitution may improve visual blurring during active head motion (14). Whitney et al. (16) recommend that “exercises to increase trunk and head movement are encouraged to improve the VOR-gain and to improve overall gait function” (16). The purpose of substitution exercises is to integrate visual and somatosensory information with vestibular input to improve gaze and postural stability (51). The rehabilitation program may include substitution exercises that alter somatosensory cues, for example, let the patient stand on different surfaces, i.e., foam with eyes open and closed; challenges the vestibular system by letting the patient perform activities with and without visual input; modified center of gravity exercises; and weight shifting (16). By removing or altering visual/somatosensory cues, the patient is forced to use remaining sensory–motor cues which will result in the fostering of responses by reacting on mainly vestibular cues (51).

##### Improve an Individual’s Balance and Postural Control

A comprehensive exercise program includes higher-balance activities that are practiced under supervision of a physiotherapist. Standing and walking exercises are progressed by changing the patients’ base of support and speed at which activities are performed (50). Progression to dual-task activities is incorporated within safety limits as the patients’ postural control (observed as improved functional ability) improves.

The principal investigator will treat all patients in the experimental group within the rehabilitation hospital settings and will therefore be aware of participant allocation. Due to the fact that rehabilitation is a multidisciplinary team approach, the patients who participate in the clinical trial will continue to be treated by other members of the multidisciplinary team, namely, the occupational therapist, the speech and language therapist, social worker, and psychologist as it is customary in the relevant rehabilitation hospital settings.

#### Control Group

The task-specific approach to rehabilitation is the standard of care intervention approach (10). An example of the flow of a therapy session based on the task-specific approach (12, 52) is described in Table 3.

**Table 3:** **An example of the flow of a therapy session based on the task-specific approach (12, 52)**.

Steps followed	Task-specific activities
Step 1	Identify the impairments and lacking components during the execution of activities
	Determine the appropriate steps that need to be facilitated to be able to complete the original task
Step 2	Choose a number of skills during each treatment that are specific to the deficits and missing components identified in Step 1 and that share similar performance components with the functional tasks trained in the same session
Step 3	Practice the skills and reinforce the practice of the missing components throughout the treatment session
Step 4	Transfer the skills practiced in Step 2 and Step 3 to practice the functional tasks in accordance with the level of balance function of the participant

Patients in Group 2 will be treated by qualified physiotherapists within the rehabilitation centers. These therapists will use the same task-specific principles in the rehabilitation of post-stroke patients as the researcher. The researcher will ensure the physiotherapists who are employees of the different rehabilitation settings are familiar and implement the same task-specific principles as the researcher treating the experimental group.

**Figure 1:**
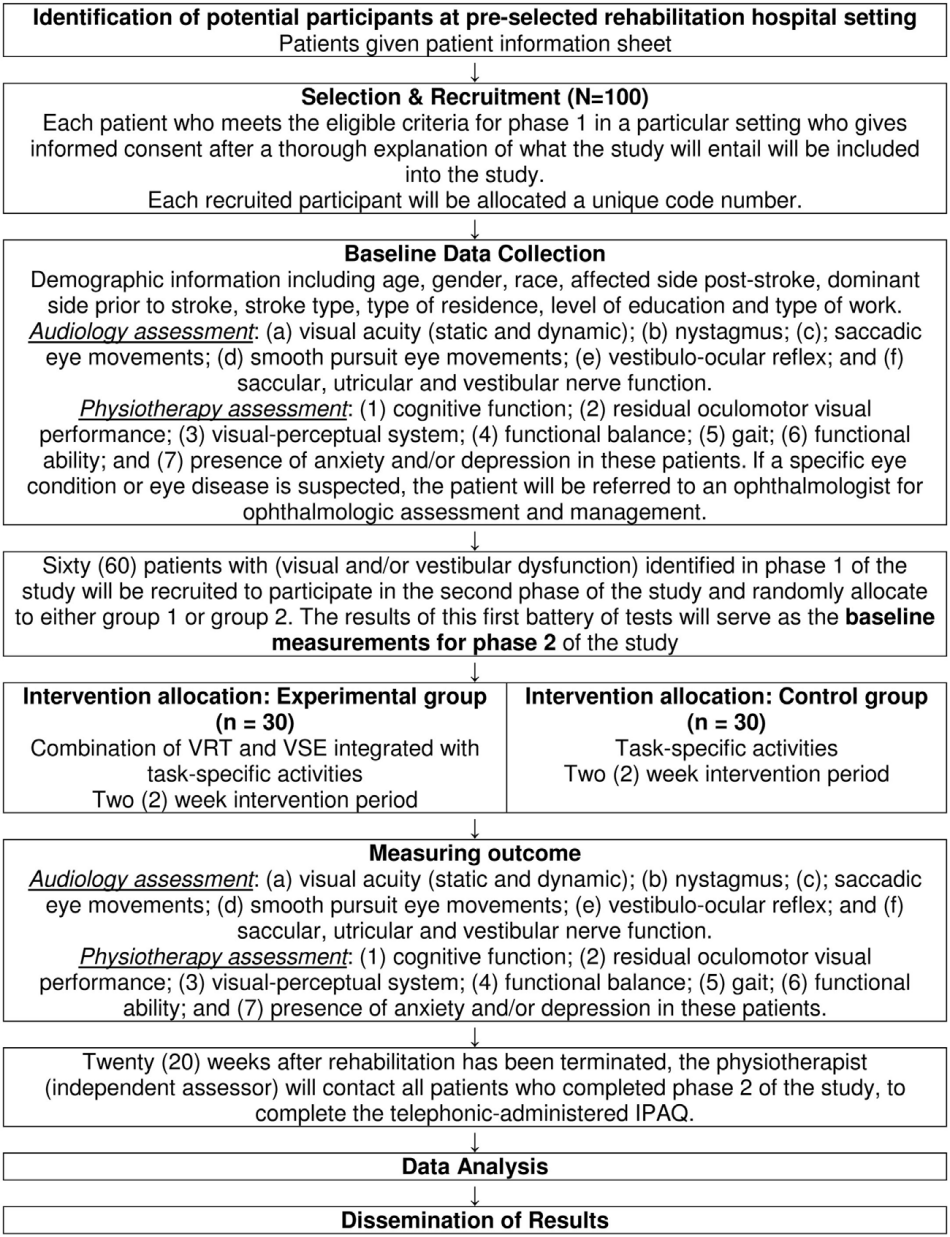
**Flow chart for study**.

The course of the study is displayed in Figure 1.

### Quality Control (Bias Protection)

All tests, completed by the audiologist and outcome measures and the physiotherapist, are internationally recognized and validated tests and outcome measures. The results of this trial may therefore be compared with studies where similar data collection methodology has been applied on both national and international level. To prevent selective assessment bias, the two independent assessors (a qualified audiologist and physiotherapist) will be blind with regard to the group that the patients will be assigned to. The physiotherapist who will conduct the assessments will be an independent assessor and not the principal investigator. The patients will be blind to the group they are assigned to. The researcher will ensure the physiotherapists treating the control group are familiar and implement the same task-specific principles as the researcher treating the experimental group. The principal investigator completed a workshop on Good Clinical Practice Guidelines presented by the UP in July 2015 (GCP 2015/07-002).

### Data Management and Analysis

Data of the cross-sectional survey (phase 1 of the study) will be analyzed and reported according to the STROBE checklist. Data of the cross-sectional clinical trial (phase 2 of the study) will be analyzed and reported according to the CONSORT statement. The outcomes measures measuring the clinical outcomes the data summary will make use of descriptive statistics, geometric mean, SD, median, range, and 95% CIs will be used when data conforms to a Gaussian distribution. Results from the two groups will be compared with respect to outcome at 2 weeks, adjusted for baseline values, using analysis of covariance (ANCOVA), using ranks when data are skewed. Data characteristics permitting, groups may also be compared using a mixed-model approach which will indicate the data over all the time points where treatment, time, and treatment–time interaction will be entered into the model, and the treatment–time interaction will be of primary intent. Testing will be done at the 0.05 level of significance.

### Ethics Considerations and Dissemination

Ethics approval has been obtained from the Ethics Committee of the Faculty of Health Sciences at the UP (374/2015) after approval of the research protocol by the post-graduate committee of the School of Healthcare Sciences, UP. The study has been registered at the Pan African Clinical Trials Registry (PACTR201509001223262). The fact that patients in Group 1 will receive intervention (VRT and VSE integrated with the task-specific activities) that patients in Group 2 will not receive, can ethically be accounted for because VRT and VSE integrated with the task-specific activities is not part of the present standard rehabilitation protocol that patients receive at the rehabilitation centers. Patients in Group 2 will therefore not be deprived of treatment that they would have received in these rehabilitation hospital centers. Patients in the experimental group will receive VRT and VSE integrated with the task-specific activities as “add on” activities. A study on the effect of VSE on the outcome of functional rehabilitation revealed that the intervention (VSE integrated with the task-specific activities) is not harmful to patients in any way (4).

Patients will be informed that their rehabilitation is part of a clinical trial. The procedures will be explained to them carefully in a way that they will understand. An information sheet and informed consent form with the clear explanation of what the study entails will be given to all prospective patients prior to commencement of the study. The patients will be provided with all information necessary to make a well-informed decision. The patient will also be informed that involvement in the study is voluntary, and she/he may leave the study at any time. The withdrawal from the study will not affect patients’ treatment at the rehabilitation center that they were admitted to. Information on each patient will be kept confidential. The information obtained will under no circumstances be disclosed to any other parties than the principal investigator and the research supervisor. Information obtained during the study will only be used for research purposes. Each informed consent form will be signed by the participant or a thumbprint if the participant is unable to write and to witnesses. The patients will only be identified by a code number allocated to them at the beginning of their participation in the study. This number will be entered into the data sheets to ensure complete confidentiality. Data will be stored on the electronic data management system of the UP, Alfresco, for a period of 15 years.

The study will be completed by the principal investigator and will be submitted as fulfillment for the PhD degree at the UP. The completed research articles will be submitted for the publication in peer-reviewed professional journals nationally and internationally. The results of this study will also be presented at various national and international congresses.

Training or/reporting to staff members at the different rehabilitation hospital settings on the integration of VSE and VRT into task-specific activities in rehabilitation will be done if the outcome of the experimental group is clinically and statistically significantly better than the results of the control group. If requested, a copy of the research article/the outcome of the study will be made available to all patients of the study. Patients who were allocated to the control group will receive the opportunity to receive VRT and VSE integrated with the task-based treatment approach received by the experimental group.

### Strengths and Limitations of this Study

#### Strengths of the Study

• A multicentre cross-sectional survey and cross-sectional clinical trial will be combined.
• Assessment of *N* = 100 stroke patients in the sub-acute phase will be completed at private and public rehabilitation settings in Johannesburg and Pretoria (multicentre).
• All tests completed by the audiologist and outcome measures and the physiotherapist are internationally recognized and validated. The results of this study may therefore be compared with similar studies where the same data capturing methods or outcomes measures have been implemented on both national and international level.
• To prevent selective assessment bias, the two independent assessors (a qualified audiologist and physiotherapist) will be blind with regard to the group that the patients will be assigned to.
• The physiotherapist who will conduct the assessments will be an independent assessor and not the principal investigator.
• The patients will be blind to the group they are assigned to.
• First inter-disciplinary study conducted by the Department of Physiotherapy and Department of Audiology at the University of Pretoria (UP), South Africa.
• Equipment that will be used during the duration of the study will be provided by “Interacoustics” and specifically the “EyeSeeCam vHIT” will be utilized in a research project for the first time in South Africa.
• The proposed study is a “follow-up” study of a previously published research: Van Wyk et al. (12).

#### Limitations of the Study

• Upon completion of the previous study by the authors, a large number of drop out of participants after discharge from various rehabilitation settings in South Africa was observed limited long-term follow-up despite the fact that participants received remuneration for traveling costs and were contacted telephonically on a regular basis. The large loss to follow-up prevented the researcher from determining whether the long-term effect of the treatment was sustained and was regarded as a limitation of the previous study.
• Twenty weeks after rehabilitation has been terminated, the physiotherapist (independent assessor) will contact all patients who completed phase 2 of the study, to complete the Telephonic-Administered International Physical Activity Questionnaire (IPAQ) to determine the effect of the combination of VRT and VSE integrated with task-specific activities for patients in Group 1 (experimental group), compared with patients in Group 2 (control group) who will receive task-specific activities alone on their participation activities 20 weeks after rehabilitation has been terminated.

## Author Contributions

AW and Dr. CE participated in the design, coordination, and drafting of the manuscript. Prof. PB performed the statistical analysis of the sample size and data analysis. Dr. BH is a co-supervisor for the study and participated in the design of the vestibular function protocol for post-stroke patients in the sub-acute phase. She has published in the field of vestibular and neurotology and has extensive experience in vestibular testing techniques, both execution thereof and data interpretation.

## Conflict of Interest Statement

The authors declare that the research was conducted in the absence of any commercial or financial relationships that could be construed as a potential conflict of interest.
